# Leptospirosis infection in a homeless patient in December in Tokyo: a case report

**DOI:** 10.1186/s13256-015-0687-4

**Published:** 2015-09-16

**Authors:** You Me Kang, Akiyoshi Hagiwara, Tatsuki Uemura

**Affiliations:** Department of Emergency Medicine and Critical Care, National Center for Global Health and Medicine, 1-21-1 Toyama, Shinjuku-ku, Tokyo 162-8655 Japan

**Keywords:** *flaB*, Homeless, Leptospirosis, Tokyo, Weil’s disease

## Abstract

**Introduction:**

We report a case of severe leptospirosis that occurred during winter in Tokyo, the capital of Japan. Leptospirosis is endemic in tropical regions and extremely rare in the urban areas of Japan. Only six new cases were reported in Tokyo in 2014. Most leptospirosis cases reported in urban areas of Japan were a result of occupational hazards, and there is no previous report of leptospirosis in a homeless patient in Tokyo. We believe this report could provide a widened perspective about the clinical presentation and epidemiology of leptospirosis in Japan.

**Case presentation:**

Our patient was a 73-year-old Asian man. He had been homeless for over 10 years, with exposure to rodents and their excrement in parks and on the streets. He presented with fever and severe inflammatory response, satisfying the diagnostic criteria for systemic inflammatory response syndrome. Laboratory findings showed multiple-organ dysfunction, including renal failure, liver failure with increased total bilirubin level, and coagulopathy with decreased platelets. We suspected leptospirosis on the basis of these clinical findings. The diagnosis was also confirmed by polymerase chain reaction first, and paired antibody titers on day 9, in the recovery period, showed positive results for three species.

**Conclusions:**

Our patient’s case suggests that even patients without a history of traveling abroad or exposure to freshwater can develop leptospirosis in winter in urban areas in Japan. If a patient has symptoms like fever, calf pain and MOF; as a differential diagnosis we should rule outthe Leptospirosis. From the perspective of sensitivity, specificity, and clinical convenience, polymerase chain reaction could be the preferred diagnostic tool of choice.

## Introduction

Leptospirosis is a zoonotic disease caused by *Leptospira*, a Gram-negative aerobic bacterium belonging to the Spirochaetaceae family. *Leptospira* can survive multiple months in freshwater or wet soil. The infectious cycle is maintained in nature by chronic infection of the renal tubules of maintenance hosts [1], the most important of which are small mammals. Symptoms of leptospirosis are highly variable, and the disease can manifest as fever, chills, headache, myalgia, abdominal pain, conjunctival suffusion, and rash, among others [2]. The great majority of infections caused by leptospires are either subclinical or of very mild severity, and patients are unlikely to seek medical attention [3]. However, the severity of leptospirosis can vary greatly, ranging from mild, flu-like illness to fatal hemorrhagic forms with severe involvement of vital organs such as the liver, lungs, and kidneys [4]. These cases can lead to multiple organ failure or other complications and may require hemodiafiltration [5] or mechanical ventilation [6]. We encountered a patient with Weil’s disease (severe leptospirosis) who was thought to have been infected in unspecified public spaces in Tokyo in December. Generally, most cases of leptospirosis in Japan occur in Okinawa, located in the southern area of Japan, via exposure to freshwater. Leptospirosis in urban areas in Japan has been thought to be extremely rare, and there has been no previous report in the literature of a homeless patient with leptospirosis. In this report, we describe a recent case of a patient with Weil’s disease in which the patient was thought to have been infected in the public spaces in Tokyo.

## Case presentation

A 73-year-old Asian man was transferred to our hospital complaining of malaise and inability to move. His past medical history included tuberculosis in childhood, but at the point of admission he was not being treated with any medication. He had been living as a homeless man for more than 10 years in Tokyo, where he had been eating food discarded from restaurants around Shibuya and Nakano and had been staying in public parks such as Jingu-Gaien during the night. He recalled that rodents such as rats had been present around his places of residence. He had not been eating well up to a few days before hospitalization.

His physical examination revealed that his height was 157cm and his body weight was 48.6kg. His level of consciousness, based on the Glasgow Coma Scale, was E4V4M6. His axillary temperature was 38°C, his blood pressure was 127/64mmHg, his respiratory rate was 22 breaths/min, and his heart rate was 102 beats/min. He reported tenderness in bilateral lower limbs. His left lower calf was red and warm compared with the right side. Other than his impaired level of consciousness, no significant findings were apparent during his neurological examination.

His blood test results were aspartate aminotransferase, 272IU/L; alanine aminotransferase, 153IU/L; lactate dehydrogenase, 675IU/L; creatine kinase, 9048IU/L; blood urea nitrogen, 122.9mg/dl; creatinine, 5.11mg/dl; C-reactive protein (CRP), 16.02mg/dl; sodium, 135mEq/L; white blood cells, 15,000/μl; hemoglobin, 10.9g/dl; platelets, 3.9×10^4^/μl; and fibrinogen, 814mg/dl. Other test results are shown in Table 1. The remainder of the examination results were normal, such as blood levels of potassium, prothrombin time and international normalized ratio, activated partial thromboplastin time, and fibrin degradation products. Chest radiography and abdominal contrast-enhanced computed tomography showed nothing significant.

**Table 1:** Laboratory findings

	Patient’s values	Normal laboratory values
Blood cell count		
White blood cells	15,030/μl	4000–9000/μl
Red blood cells	3.66×10^4^/μl	4.1–5.3×10^4^/μl
Hemoglobin	10.9g/dl	14–17g/dl
Hematocrit	30.3%	39–52%
Mean corpuscular volume	82.8fl	84–95fl
Mean corpuscular hemoglobin	29.8pg	27–32pg
Mean corpuscular hemoglobin concentration	36g/dl	32–36g/dl
Platelets	3.9×10^4^/μl	1.5–3.5×10^4^/μl
Neutrophils	95.9%	48–61%
Lymphocytes	2.6%	25–45%
Monocytes	1.4%	4.0–7.0%
Eosinophils	0%	1.0–5.0%
Basophils	0.1%	0.0–0.1%
Coagulation		
Prothrombin time	11.9s	9.8–12.4s
International normalized ratio	1	0.9–1.16
Activated partial thromboplastin time	28.3s	26.5–38.1s
Fibrinogen	814mg/dl	130–316mg/dl
FDP	8.9μg/ml	1.5–3.5μg/ml
Biochemistry		
Albumin	3.2g/dl	3.9–5.1g/dl
Total bilirubin	1.3mg/dl	0.2–1.2mg/dl
AST	272IU/L	13–33IU/L
ALT	153IU/L	8.0–42IU/L
LDH	675IU/L	119–229IU/L
ALP	331IU/L	115–359IU/L
γ-GTP	62IU/L	10–47IU/L
Creatine kinase	9048IU/L	62–287IU/L
Blood urea nitrogen	122.9mg/dl	8.0–20mg/dl
Creatinine	5.11mg/dl	0.61–1.04mg/dl
eGFR	9.5	ml/min/1.73m2
Cl^−^	93mEq/L	98–108mEq/L
Na^+^	135mEq/L	135–145mEq/L
K^+^	3.9mEq/L	3.6–5.0mEq/L
Ca^2+^	8mg/dl	8.6–10.1mg/dl
Blood sugar	132mg/dl	70–109mg/dl
C-reactive protein	16.02mg/dl	0.0–0.3mg/dl
Blood gas analysis (venous blood, room air)		
pH	7.44	7.35–7.45
PaCO_2_	30mmHg	35–45mmHg
Base excess	−3.8mmol/L	−2mmol/L to 2mmol/L
HCO_3_^−^	20.4mmol/L	22–26mmol/L
Blood sugar	127mg/dl	70–109mg/dl
Lactate	1.4mmol/L	0.5–2.0mmol/L
Urinalysis		
pH	5	5.0–7.0
Urine-specific gravity	1.013	1.005–1.030
Protein	1+	–
Occult blood	3+	–
White blood cells	+/−	–

*ALP* alkaline phosphatase, *ALT* alanine aminotransferase, *AST* aspartate aminotransferase, *CRP* C-reactive protein, *eGFR* estimated glomerular filtration rate, *FDP* fibrin degradation products, *γ-GTP* γ-glutamyl transpeptidase, *LDH* lactate dehydrogenase, *PaCO*_*2*_ partial pressure of carbon dioxide

**Fig. 1:**
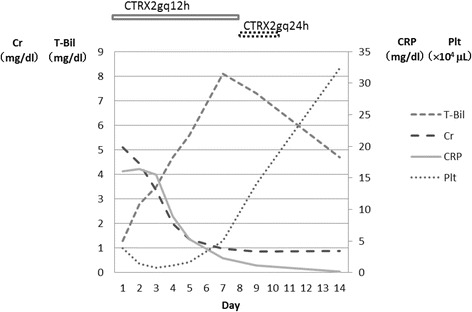
The patient’s clinical course. His total bilirubin (T-Bil) continued to increase, reaching 8.1mg/dl on day 7 and peaking after that. His serum creatinine (Cr) was 5.1mg/dl on the day of admission and subsequently showed continuous improvement. His C-reactive protein (CRP) level was 16.02mg/dl on day 1 but continued to decrease and was within the normal range by day 12. His platelet (Plt) count was 3.9×10^4^/μl on the day of admission, reaching a minimum value of 0.8×10^4^/μl on day 3. After that, his platelets started to increase and normalized by day 9. Ceftriaxone (CTRX) was initially administered at 2 grams every 12 hours, with the dosage changed on day 8.

The clinical course of the patient is shown in Fig. 1. His condition satisfied the diagnostic criteria for systemic inflammatory response syndrome [7]. Severe sepsis was diagnosed and he was admitted to the critical care unit. Blood, urine and sputum cultures were collected and analyzed with no significant findings. Although the laboratory test results for this patient showed marked multi-organ involvement, the constellation of characteristic features of high fever, lower calf pain, elevated white blood cell count, elevated CRP level, acute renal failure, liver failure and thrombocytopenia suggested a strong possibility of leptospirosis than other etiologies. The patient also had a history of exposure to rodents and their excrement, so we considered leptospirosis as the first diagnostic option and started antibiotic therapy with ceftriaxone (4g/day). On day 2, fever and redness and warmth of the left lower calf disappeared. On day 3, maximal axillary temperature was 37°C, and the patient did not subsequently present with fever.

In contrast, the patient’s thrombocytopenia progressed, and his platelet count on day 3 was 8000/μl. Although his platelet count was low, no clinical signs of bleeding were identified. Because thrombocytopenia in leptospirosis is transient and does not result from disseminated intravascular coagulation [8], we decided not to perform a transfusion immediately. Recovery was seen starting from day 4 and had normalized by day 9. His total bilirubin level continued to rise, reaching 8.1mg/dl on day 7 before starting to decrease, and reaching 4.7mg/dl on day 14.

Regarding renal failure, 24-h urine collection was performed to evaluate the cause. His fractional excretion of sodium was 7.91%, his osmotic pressure of urine was 383mOsm/kg, and his total urine output was 3970ml/day. Adequate fluid was administered, and his creatinine level normalized to 0.98mg/dl on day 7. On day 2, hypokalemia was found in laboratory testing and was attributed to non-oliguric renal failure. We continued to administer potassium intravenously (20–40mEq/L/day) until day 5, when his potassium level reached 3.0mEq/L. During the admission, there was no compliance issue. On day 16, the patient was discharged without major complications.

Serum and urine samples taken on hospital days 2 and 9 were subjected to microscopic agglutination testing and polymerase chain reaction (PCR) testing. In the PCR, we used a portion of the *flaB* gene coding for the flagellar protein of leptospires. Microscopic agglutination testing was performed with a panel of 15 live leptospires representing the main serovars commonly reported in Japan. Antibody titers against 16 *Leptospira* spp. in the serum sample from hospital day 2 were all under 50U/ml. However, PCR with serum and urine specimens yielded positive results, confirming the diagnosis of leptospirosis. We were later notified that titers of antibodies against *L. borgpetersenii* serovar Poi, *L. interrogans* serovar Hebdomadis, and *L. interrogans* serovar Kremastos in the serum sample from hospital day 9 were elevated to 400–1600 U/ml (Table 2).

**Table 2:** Microscopic agglutination test results

Antigens		Agglutination titers, U/ml	
Species	Serovar	Hospital day 2	Hospital day 9
*Leptospira borgpetersenii*	Castellonis	<50	<200
*Leptospira borgpetersenii*	Javanica	<50	<200
*Leptospira borgpetersenii*	Poi	<50	1600
*Leptospira interrogans*	Australis	<50	<200
*Leptospira interrogans*	Autumnalis	<50	<200
*Leptospira interrogans*	Bataviae	<50	<200
*Leptospira interrogans*	Canicola	<50	<200
*Leptospira interrogans*	Copenhageni	<50	<200
*Leptospira interrogans*	Hebdomadis	<50	400
*Leptospira interrogans*	Icterohaemorrhagiae	<50	<200
*Leptospira interrogans*	Kremastos	<50	400
*Leptospira interrogans*	Pomona	<50	<200
*Leptospira interrogans*	Pyrogenes	<50	<200
*Leptospira interrogans*	Rachmati	<50	<200
*Leptospira interrogans*	Grippotyphosa	<50	<200

## Discussion

We suspected leptospirosis in this case on the basis of the constellation of characteristic features and laboratory results. Therefore, we could administer an appropriate antibiotic option starting from day 1 and collect specimens for specific tests. Thrombocytopenia, acute renal failure, elevated total bilirubin, and severe inflammatory reaction are not uncommon features of severe leptospirosis previously reported in Japan, and several cases required transient hemodialysis owing to uremic symptoms.

Severe cases of leptospirosis, or *Weil’s disease*, are treated using antibiotics such as penicillin G, cefotaxime, doxycycline, and ceftriaxone [9]. Reports have suggested that ceftriaxone and sodium penicillin G are equally effective for the treatment of severe leptospirosis [10]. We therefore selected ceftriaxone as the first-line treatment option. As a result, the condition of the patient responded very well and the therapeutic results were satisfying.

The diagnosis of leptospirosis can be made on the basis of culture, serology, and PCR. Leptospires require delicate and complex culture media. The media must contain several growth-promoting substances and selected antimicrobial agents to suppress contaminants. Culturing leptospires is thus laborious, expensive, and tedious [11]. The serological method, however, is neither sensitive nor specific during the first week of illness [12], and paired serum samples are required for serological diagnosis in patients such as ours. In our patient, we performed both PCR and serology tests. PCR confirmed the diagnosis of leptospirosis first, followed by antibody titers during the recovery period that showed positive results for three species.

## Conclusions

Only six new cases of leptospirosis were reported in Tokyo in 2014 [13]. Several case reports and a literature review about leptospirosis in Japanese urban areas have been published. This case report is the first, to the best of our knowledge, about leptospirosis in a homeless patient in an urban area of Tokyo. Major causes of infection in previous reports were occupational hazards or continuous contacts with rats inside the patients’ own houses [14]. Leptospirosis in the present case was probably caused by exposure to urine from rats inhabiting the parks and streets of Tokyo. This suggests that rodents inhabiting public spaces in Tokyo represent an infectious reservoir for leptospirosis. In addition, this case arose in December, a winter month in Japan, even though most cases of leptospirosis in Japan are typically encountered in summer.

Leptospirosis should not be ruled out in patients with characteristic symptoms, signs, and laboratory results, even in the absence of a history of travel abroad or exposure to freshwater. In addition, PCR offers an effective and quick diagnostic method when serological diagnostic methods cannot confirm the diagnosis. However, to ensure a high sensitivity, samples have to be obtained before or shortly after the start of antibiotic therapy because antimicrobial agents quickly remove *Leptospira* from the blood [15].

## Consent

Written informed consent was obtained from the patient for publication of this case report and accompanying images. A copy of the written consent form is available for review by the Editor-in-Chief of this journal.

## Abbreviations

ALP: alkaline phosphatase
ALT: alanine aminotransferase
AST: aspartate aminotransferase
Cr: creatinine
CRP: C-reactive protein
CTRX: ceftriaxone
eGFR: estimated glomerular filtration rate
FDP: fibrin degradation products
γ-GTP: γ-glutamyl transpeptidase
LDH: lactate dehydrogenase
PaCO_2_: partial pressure of carbon dioxide
PCR: polymerase chain reaction
Plt: platelets
T-Bil: total bilirubin
